# A Joint Model Provisioning and Request Dispatch Solution for Low-Latency Inference Services on Edge

**DOI:** 10.3390/s21196594

**Published:** 2021-10-02

**Authors:** Anish Prasad, Carl Mofjeld, Yang Peng

**Affiliations:** Division of Computing and Software Systems, University of Washington Bothell, Bothell, WA 98011, USA; anp10@uw.edu (A.P.); cmofjeld@uw.edu (C.M.)

**Keywords:** machine learning inference, edge computing, Kubernetes

## Abstract

With the advancement of machine learning, a growing number of mobile users rely on machine learning inference for making time-sensitive and safety-critical decisions. Therefore, the demand for high-quality and low-latency inference services at the network edge has become the key to modern intelligent society. This paper proposes a novel solution that jointly provisions machine learning models and dispatches inference requests to reduce inference latency on edge nodes. Existing solutions either direct inference requests to the nearest edge node to save network latency or balance edge nodes’ workload by reducing queuing and computing time. The proposed solution provisions each edge node with the optimal number and type of inference instances under a holistic consideration of networking, computing, and memory resources. Mobile users can thus be directed to utilize inference services on the edge nodes that offer minimal serving latency. The proposed solution has been implemented using TensorFlow Serving and Kubernetes on an edge cluster. Through simulation and testbed experiments under various system settings, the evaluation results showed that the joint strategy could consistently achieve lower latency than simply searching for the best edge node to serve inference requests.

## 1. Introduction

In the Internet of Things (IoT) era, inference services on edge are vital for mobile IoT devices with limited computing power yet require computationally intensive decision-making to fulfill their purpose. Intelligent processing, or machine learning-based inference, has become instrumental in tackling this issue. Traditionally, machine learning (ML) is carried out in the cloud with hundreds of server blades, which offer high-performance levels for the training and inference that ML applications require [1]. However, a cloud-centric operating paradigm may not suit many emerging mobile applications. For example, people with vision or hearing disabilities may rely on intelligent cognitive devices to help them avoid obstacles. For these emerging applications to be useful, machine learning inference will need to be carried out quickly and within close proximity to mobile users [2]. This new operating paradigm has given rise to the idea of mobile inference serving at the network edge [3].

When edge nodes are deployed in high-traffic public spaces and cellular towers [4], mobile users can offload computing-intensive inference jobs [5,6] to these edge nodes instead of running them on their own computationally limited devices. An essential requirement for edge-based inference services is that machine learning models must be provisioned such that they are available close to a user when the user needs inference services. Taking advantage of proximity to the client is the primary method that edge ML solution uses to offer performance comparable or superior to cloud-based solutions [7].

Traditional solutions leveraging edge nodes for inference serving strive to reduce the serving latency by focusing on the following techniques, each with unique benefits and drawbacks. Some solutions focus on balancing the performance of the edge nodes by distributing similar workloads across multiple identically provisioned nodes so that queuing and computing delay can be reduced across the entire edge cluster. However, this solution may increase the serving latency since it does not compensate for a mobile client’s movement. On the other hand, some solutions prefer to select edge nodes based on the user’s physical proximity to the edge node and assume that the closest node (in network distance) will have the least latency. However, it is possible that the edge node closest to a mobile user may not be the one that will respond with minimal inference latency. Multiple factors such as the current deployments on the edge nodes, current memory utilization, and current workload can affect the overall serving latency. An alternative solution is the container migration technique [8,9], which provisions the required inference services when a request arrives or when the user moves to a new edge node. Architectures that support container migration include uncoordinated [10], orchestrator-coordinated [11], and user-driven [12]. Container migration is important when successful inference requires contextual or historical information to be preserved when moving execution between edge nodes. However, a migration-based solution is untenable since both migration and provisioning may take an unpredictable amount of time, worsening under load from numerous concurrent requests.

This paper considers the limitations of existing solutions and presents a joint model provisioning and request dispatch solution for serving mobile inference requests at the network edge. The key idea is to provision an edge node with the inference resources most likely to be requested and direct a user to the optimal node to service their request. This way, both communication and computing latency can be reduced. As a scheme for managing the deployment and execution of ML inference on distributed edge nodes, our solution brings together multiple elements into a cohesive system to achieve the design goal of reducing inference serving latency.

The contributions of this work are summarized below.

- This work studied a new problem in mobile edge computing, namely, joint inference model provisioning and request dispatch. This problem has been formally presented as a Non-Linear Programming (NLP) problem.
- This work implemented a system solution with front-end, back-end, and NLP solver modules to accomplish the design goal. The solution’s software modules are fully containerized and can be ported to various container-friendly platforms.
- The solution has been evaluated through testbed and simulation experiments. The results demonstrated that the proposed solution could effectively reduce the inference serving latency compared to existing solutions.

The rest of this paper is organized as follows. Section 2 formally presents the core problem, while Section 3 delves into the design and implementation details of the proposed solution. Section 4 analyzes the evaluation results obtained through simulation and testbed experiments. Section 5 discusses related work. Finally, Section 6 concludes this paper.

## 2. Analysis

Our solution aims to minimize the inference serving latency through jointly provisioning ML models and dispatching requests. This section formally presents the solution’s core problem and system model.

### 2.1. System Model

The logical system under consideration consists of three key components: requests, edge nodes, and a provisioner.

- A request denoted as $r_{i}$, is generated by a mobile user for some inference job (machine learning model). Different requests may use the same model for inference. After communicating with a nearby edge node, a user decides which node to execute its inference jobs on. *N* represents the total number of requests.
- An edge node, denoted as $s_{i}$, executes an inference job upon request. *S* represents the total number of edge nodes. An edge node has limited memory, meaning it can only host a limited number of model instances at any given time. Nevertheless, it can adjust which ML models are available based on the provisioner’s decisions.
- The provisioner determines what machine learning models will be provisioned on an edge node. The provisioner runs optimization algorithms to find the best node for each request to minimize the overall inference latency, including communication and computing time.

### 2.2. Problem Statement

Suppose an edge network has been deployed, with multiple mobile users requesting inference services. We can formally define the model provisioning and request dispatch problem based on the system model.

- Objective:
  − min{U}, where $U=\frac{\sum_{i=1}^{N}\sum_{j=1}^{S}I_{i,j}\left(\tau_{i,j}+\theta_{i,j}\right)}{N}$
- Given:
  − $\theta_{i,j}$: execution time of request $r_{i}$’s inference job on edge node $s_{j}$
  − $\tau_{i,j}$: round-trip communication time between request $r_{i}$’s originating user and edge node $s_{j}$
  − $m_{i}$: memory requirement of the model requested by $r_{i}$
  − $M_{i}$: available memory resources on edge node $s_{i}$
- Output
  − $I_{i,j}$: indicator that requests $r_{i}$ executes on edge node $s_{j}$
- Subject to:
  − Memory constraint:
    for each edge node $s_{j}$, $\sum_{i=1}^{N}I_{i,j}m_{i}⩽M_{j}$
  − Edge node selection constraint:
    * for each request $r_{i}$, $\sum_{j=1}^{S}I_{i,j}⩾1$
    * $I_{i,j}=\left\{0,1\right\}$

In this formulation, the optimization objective *U* represents the average serving latency of all requests. The output $I_{i,j}$ is an integer value, which is used to direct user requests and provision models. Each request $r_{i}$ must be routed to some edge node. When a request is routed to execute on node $s_{j}$, $I_{i,j}=1$, otherwise, $I_{i,j}=0$. Although the edge node selection constraint may allow a request to be scheduled to multiple edge nodes, the optimal solution will only select one node for the lowest latency. The provisioner also uses this indicator to deploy ML models on selected edge nodes.

Based on this formulation, Section 3 will present the design and implementation details about how to collect input data and execute the outputs of this NLP-oriented problem in the proposed solution.

## 3. System Design and Implementation

### 3.1. System Overview

**Figure 1:**
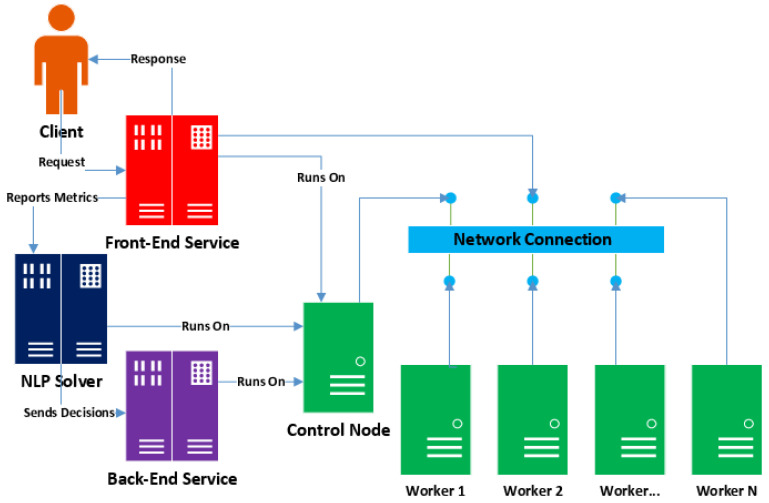
Overview of the system.

Figure 1 depicts a simplified architecture of the proposed system, which consists of three logical software modules: a front-end service, a back-end service, and a Non-Linear Programming (NLP) solver. The front-end service is designed to handle user requests, while the back-end service provisions inference instances (containers consisting of models and the needed environment components) and executes inference jobs. The NLP solver acts as the bridge between the front-end and back-end services. This design decouples the user-facing functionality from back-end functionality to restrict each module to a specific set of operations within a defined scope.

#### 3.1.1. Front-End Design Overview

The front-end module has two primary functions that handle user requests. Firstly, it responds to the user’s inference requests with the optimal endpoint (IP address and port number). Secondly, it is responsible for collecting metrics related to inference request patterns. The metrics are made accessible through back-end facing REST endpoints. This design was chosen primarily to enable the front-end and back-end services to operate independently. This means the front-end will continue to function regardless of what resource provisioning solution runs at the back-end.

#### 3.1.2. Back-End Design Overview

The back-end module’s core function is performing real-time provisioning operations. It uses the NLP solver to decide where to provision ML models. When making these optimization decisions, the back-end module has two operating modes (see Section 3.2 for details) to allow for an implementation that can prioritize either short-term serving latency or long-term system stability. The back-end module also handles the deletion of inference instances and the actual execution of inference jobs. Similar to the front-end, the back-end does not depend on a specific front-end service to be running as long as the data required is provided by the front-end.

#### 3.1.3. Non-Linear Programming Solver Overview

The NLP solver is arguably the system’s most crucial component, which solves an optimization problem given performance requirements and system constraints. It forms a key connection between the other two modules by taking the front-end’s data and offering the back-end provisioning decisions. The back-end service uses these decisions to provision models, which affects how the front-end service can handle user requests, creating a cycle through which the system can continuously modify current deployments for better inference serving.

### 3.2. System Implementation

In our implementation, the system’s front-end and back-end services run within Docker atop a Kubernetes cluster [13]. The NLP solver runs directly on the control node hardware.

- The front-end service is a RESTful API implemented with Python Flask [14]. Its main function is accepting user requests and returning either a specific endpoint or a list of endpoints, proxying requests, and collecting metrics to be used by the back-end service.
- The back-end service is also written in Python. It invokes the NLP solver with metrics data obtained by the front-end service, and issues commands through the Kubernetes Client Library to perform the actual provisioning tasks based on the solver’s decisions.
- The NLP solver is implemented using the Ipopt solver [15] from the COIN-OR suite [16]. Ipopt is used in conjunction with AMPL (a modeling tool for solving NLP problems [17]) to allow for dynamic generation of configuration files based on templates when system conditions change.

In the current implementation, ML models are served using TensorFlow Serving (TFS) containers [18]. TFS is employed because its design allows user access through standard REST API formats. TensorFlow Serving’s community also offers pre-compiled Docker images that can be run in Kubernetes within various environments.

#### 3.2.1. Front-End Service Implementation Details

**Figure 2:**
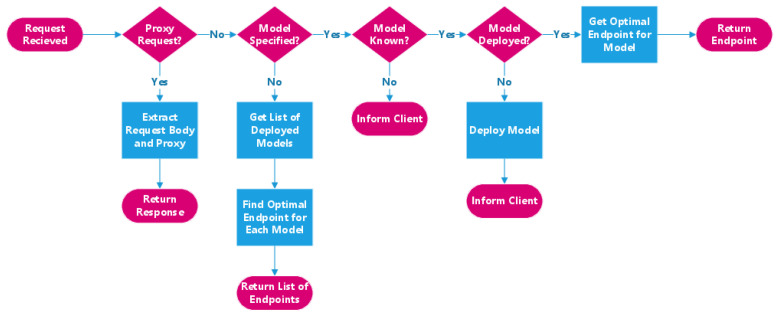
Overview of the front-end execution path.

The front-end service is designed around a Python Flask server [14]. The Flask server defines the set of five APIs available to the front-end service in five Python functions. There are two user-facing endpoints (the service query and service proxy endpoints, respectively). Additionally, three back-end facing endpoints were implemented to allow the back-end to acquire statistics, such as when and how often a given model is requested, stored locally by the front-end. The system invokes the correct function to handle the request based on the parameters included within the request endpoint URL. Once routed to the correct function, the Flask server parses the URL and the request body to obtain the required information to execute the function, such as the function’s name. Helper files were created to house the code defining the system’s functionality separately from the Flask server. This code defines the logic for locating a model, creating a deployment, or producing a list of all models. The Flask server invokes code from these helper files to perform its operations and then returns the result to the user in an HTTP response code. Figure 2 depicts the logical steps taken by the front-end service when handing a user request.

#### Service Query Endpoint

This endpoint is responsible for returning the user a serving endpoint for one specific model or a list of endpoints for all available models in the system. To provide this function, the service will first check if the user has included a model request in their request body. If there is a request body, the system will convert it to JSON and extract the name of the requested model from the resulting JSON object. It will then invoke the function that searches the cluster for the required models. The endpoint that this function returns is returned to the user as a response body along with an HTTP response code. If the specified model cannot be found, a 404 status is returned to the user. If no request body is specified, the system will perform the same steps for every deployed model and return a list of endpoints in the response body. As each deployed model must have a corresponding deployment in Kubernetes, the list of deployments is identical to the list of available models.

It is important to note that the system considers each model a running service irrespective of the number of replicas, meaning that each model could have an arbitrary number of replicas running. However, the system will see them all as one inference resource and pick one of these replicas to serve the user. Additionally, if the user requests a model that the system is aware of but is not currently serving, the front-end can provision the model on the cluster independent of the back-end to serve the user immediately. The next time the user requests a model that was not provisioned initially, they will receive an endpoint to the newly provisioned model. The back-end and the NLP solver will consider any models provisioned this way when they perform their routine optimization operations.

#### Service Proxying Endpoint

Proxying inference requests for some models is a more involved process than simply returning an endpoint. The user informs the front-end service that it wants a request proxied by including the TensorFlow Serving model path from the request endpoint [18]. This path is provided to the function within the request endpoint. For example, instead of just making a request on */services*, they may request */services/model1/v1/model1b/add*. The service will then route the request to a function that can handle proxying. However, instead of simply returning the endpoint to the user, the system extracts the information from the request body meant for the model from the original request. The service then performs the inference request on the model itself by performing a POST request on the model endpoint obtained through the endpoint search logic described in Section 3.2.1. The inference response is then returned to the user.

#### Back-End Facing Endpoints

While serving users, the user-facing endpoints also help collect metrics about the inference request patterns. This information is stored in text files on the local file system of the device the front-end service is running on. This information is accessible to the back-end service via three endpoints. These endpoints allow each module to remain semi-disconnected and avoid concurrency problems.

- Request Stats Endpoint. The front-end records the model requested, the access latency, and the chosen edge node for every request. The back-end uses this data to make provisioning decisions.
- Model Stats Endpoint. The front-end records the last time a model was requested and the number of times a model has been requested since it was provisioned. The back-end uses this data to remove unnecessary services.
- Node Memory Stats Endpoint. Similar to the request stats endpoint, this endpoint is used by the back-end to acquire the current RAM usage of each node in the cluster. This information is critical for deciding which node to place a model on and from which node to remove a model.

#### 3.2.2. Back-End Service Implementation Details

The system invokes the back-end service periodically (as defined by the system administrator) using collected request and cluster information. The back-end service first performs a GET request on each of the three stats endpoints to acquire the required information. The back-end is responsible for invoking the NLP solver to obtain provisioning decisions. The solver requires the optimization function and the input data to be supplied in a specific format. The back-end contains functions that use the statistics obtained from the front-end to build input text files in the format the solver expects. This is achieved by using template files with marked fields that are filled programmatically. Once that is complete, the Python *os* module is used to interact with the underlying operating system and invoke the NLP solver. When the Non-Linear Programming solver runs, it outputs an array of provisioning decisions corresponding to each model on each edge node. These decisions are then used to generate YAML files [19] for each deployment that needs to be performed. These files are used to provide the Kubernetes cluster instructions on how to deploy a model. This includes information such as the name and location of the Docker container, the number of replicas, labels that specify required hardware, and more. The logic for generating these files is similar to that used to generate files for the solver. Once the files are generated, the Kubernetes commands are issued to the cluster through the Kubernetes Client Library [20].

#### Solver Mode

As mentioned in Section 3.1, the back-end service operates in one of two modes to allow optimization of short-term latency or system stability.

**Figure 3:**
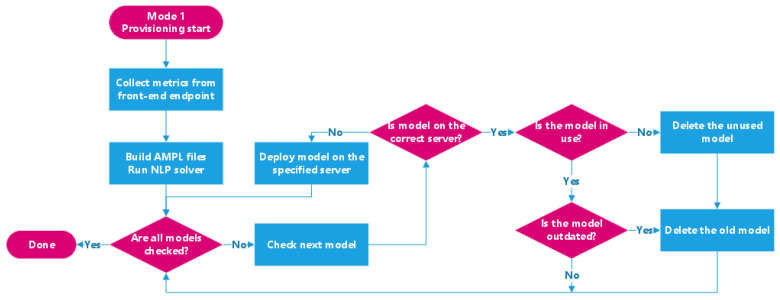
Overview of the back-end execution path in Mode 1.

**Figure 4:**
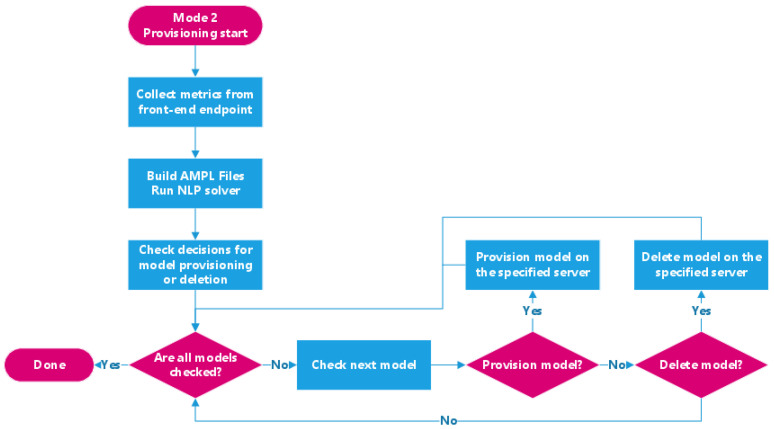
Overview of the back-end execution path in Mode 2.

- Mode 1 is the primary mode where the system prioritizes minimizing instant serving latency after provisioning. In this mode, the system provisions new models based on the decisions of the solver but does not do so when deleting models. Instead, the system deletes models based on how recently and frequently they have been requested when the memory resource becomes tight in the system. It also uses the available memory on each edge node when executing the optimization calculation. Figure 3 visualizes Mode 1 of the back-end service.
- Mode 2 optimizes the system’s performance during the stable time but at the potential cost of temporary latency increase. Instead of using the available memory on each node (which might be scarce during peak usage) for the solver’s optimization function, this mode uses each node’s total amount of memory when invoking the solver. As a result, the back-end service strictly follows the solver’s decisions when performing provisioning tasks, immediately deleting model instances if the solver does not decide to place them on an edge node. Figure 4 shows how Mode 2 operates, as well as how it differs from Mode 1.

In the current implementation, system administrators can choose the operation mode when starting the back-end services. Dynamic switching between these modes is left for future work.

#### Non-Linear Programming Solver Implementation

The NLP solver is implemented as part of the back-end service within our system, though it is logically a separate component. We used an NLP solver tool called AMPL to define an NLP optimization problem using specially formatted model and data files that define the problem’s scope. Specifically, a model file defines the optimization problem and the constraints that bind it, while a data file defines the data used in the calculations. These files are generated by Python code from a template file that defines the required format. The number of edge nodes, number of requests, latency values, the execution time of a model on a node, the amount of memory needed by a model, and the amount of available memory on each node are written into the data file. The system then generates a run file, which is simply a method of storing all of the command-line commands issued to AMPL if the solver were to be run manually. AMPL is then invoked in the command-line through the Python *os* module. Once the provisioning is complete, the system deletes any temporary files to avoid any chance that previous results might contaminate future solver runs.

#### Model Provisioning

To deploy a model, the system uses a template file containing information such as the Docker image’s location and the name of the credential file to access a private Docker repository. Using a method similar to the one used to generate files for AMPL, the system creates a new file with critical info obtained at runtime, such as the node’s name on which to deploy a model. As described in Section 3.2.2, this file is then used to invoke the deployment function via the Kubernetes Client Library. The function to create a Kubernetes service, which exposes the pods for use, is also invoked using another YAML file that defines the service parameters in the format required by Kubernetes.

It is also possible to delete a deployed model and its related service. This operation is only performed by the back-end service. The system will first request a list of pods from the cluster and check if the model specified for deletion is running. It will then use the full name of the deployment and the service to instruct the cluster to delete both using two successive commands. The cluster will then immediately delete the service and terminate the associated pods. Once complete, the front-end service will no longer find or offer the deleted model to a user.

## 4. Performance Evaluation

The proposed solution has been evaluated through both testbed and simulation experiments.

### 4.1. Testbed Experiments

**Figure 5:**
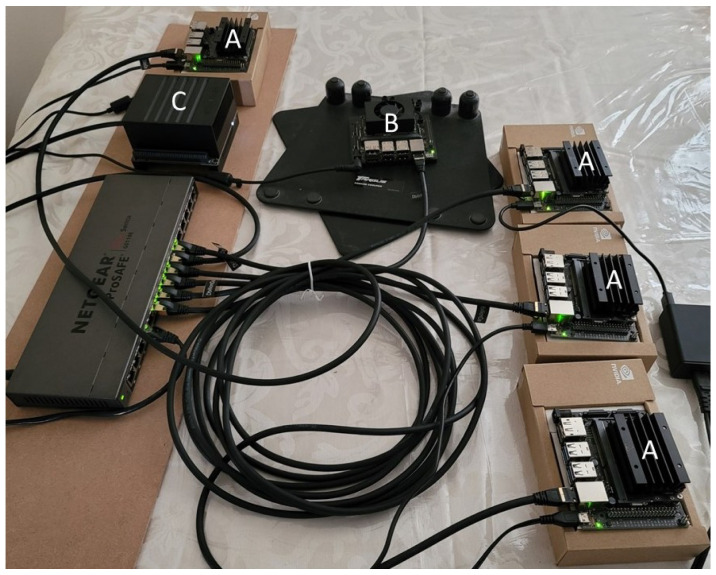
The prototype system: Devices labeled with “A” are Jetson Nanos, device “B” is the Jetson Xavier NX, and device “C” is the Jetson AGX Xavier.

To evaluate the aspects of performance that are difficult to measure in simulations, we conducted experiments in a small-scale edge cluster with six Nvidia Jetson development kits [4]. This testbed includes one Nvidia Jetson Xavier NX as a control node, four Nvidia Jetson Nano units as standard workers, and one Nvidia Jetson AGX Xavier as a more powerful worker. All devices were run with a Nvidia JetPack [21] based on Ubuntu 18.04. Figure 5 depicts the prototype system. Since Nvidia Jetson hardware is ARM-based, the official Docker images, which only support x86-based hardware, could not be used as-is. To mitigate this issue, a community-developed Docker image that can be run on ARM was used instead [22].

#### 4.1.1. Testbed Experiment Setup

This experiment was run on the prototype system under the following conditions:

- All devices in the system only run default Kubernetes programs such as Kubelet at the start. Kubelet consumes about 300 MB RAM, and it always runs when the device is active [19].
- The TensorFlow Serving Docker container [22] provides the baseline packages required to serve a TensorFlow model.
- Each model is in a docker container stored on the worker devices’ local storage. There are no models provisioned on the system before the test.
- Four models are used in the experiments, and the model container size is between 100 and 200 MB.

#### 4.1.2. Testbed Experiment Results

Aside from obtaining empirical data (such as the execution time of inference jobs) for simulation experiments to use, one important goal of the testbed experiments was to assess the system’s overhead–the provisioning latency on real hardware. As described in Section 3.1, the back-end service has two operating modes: Mode 1 delays the deletion of model instances to the system’s idle time; Mode 2 takes the opposite approach by freeing resources immediately per the solver’s output. This difference in operation means that Mode 1 should produce lower provisioning latency at the cost of more system memory being used. In contrast, Mode 2 should be the opposite.

The provisioning latency is defined as the amount of time the provisioning task is executed to its completion. It does not include the solver’s execution time. Table 1 lists the average results.

From these results, we can see that provisioning models require more time than deleting them. Provisioning one model takes more than 2500 ms, whereas deleting one takes only around 272 ms. When we perform provisioning and deletion operations simultaneously, the measured latency immediately increases. For example, two deletions and one provisioning task performed together resulted in a latency value of 3150.921 ms. As we added more models for provisioning and deletion, the overall provisioning latency values increased even further. The addition of time-consuming deletions in almost every Mode 2 execution impacts the latency by several hundred milliseconds per model to be deleted. However, the system will run better in the long term since unnecessary models have been deleted, freeing valuable memory resources. Mode 1 instead reduces the length of the unstable period when provisioning operations are in progress. Since the old services are still available, the latency experienced by the user should be reduced further since more replicas are available. Of course, this comes at the cost of available memory resources.

We further looked into the factors contributing to provisioning or deletion latency. The major factors include the load on the device, the number of replicas, the size of the model requested, and the number of required operations.

- The number of replicas plays a dominating role in the overall latency. Once the command to delete a container is received, Kubelet will instruct all corresponding pods to destroy themselves. However, the amount of time it takes for each pod to destroy itself depends on the state of the physical device it is running on. The more replicas there are, the more pods there are, and the longer it will take to delete them. The same applies to provisioning. The more replicas are requested, the more pods must be created and executed.
- The device’s current load significantly affects how long it takes to provision a model. If the CPU is busy, the provisioning of a model might take a long time, as the provisioning operation is just one out of many processes scheduled by the CPU.
- The model’s size is directly related to the amount of RAM it needs and the amount of time it takes to load the model into memory from local storage. Such loading time is typically short.
- Finally, the number of required operations also affects the provision/deletion latency. The system will execute the management commands as soon as it can. This means that multiple provisioning and deletion commands will be carried out simultaneously as the control node instructs worker nodes to perform the specified action. If multiple commands are executed simultaneously, the whole process will slow down since the hardware must split its resources among several operations.

### 4.2. Simulation Experiments

In addition to the testbed experiments, we further evaluated the proposed solution’s performance with larger-scale system settings through simulation experiments.

#### 4.2.1. Simulation Experiment Setup

The simulator was written in Python and executed on a computer with an x86-64 CPU running Ubuntu 20.04. The simulator invoked the same functions (such as generating model and data files and running the NLP solver) that were used in the testbed, while the system parameters were set according to the settings in Table 2.

In the simulations, a naive iterative search solution is also evaluated to provide a point of comparison. This naive solution searches through every edge node for minimal communication and execution latency when serving a request. The minimum latency value is recorded as the objective value for the naive solution. In other words, this naive solution does not proactively deploy model instances to the best edge node for user access; instead, it passively uses the given resources to obtain the best result.

The simulation experiments focused on assessing the average serving latency of inference requests under the influence of the following two parameters:

- The total number of requests per second,
- The total number of edge nodes.

Though other parameters may potentially affect the system performance, user requests and edge nodes constitute the most likely points of variance in the real-world implementation. Increasing the number of requests represents an increase in the system load, while increasing the number of edge nodes represents the changing hardware landscape and the available system resources to use.

For every test, the requested model is randomly picked from a pool of ten models. The round-trip communication time experienced by the user is uniformly distributed within a fixed bound, as well as all other parameters listed in Table 2. In particular, the range of round-trip communication time is obtained through empirical experiments using a local server and a remote server in the cloud. The ranges of inference execution time and inference instance size are set based on the testbed experiments described in Section 4.1. The ranges of available system memory and total system memory are set according to the specification of two typical small edge devices used in literature—Nvidia Jetson series [4] and Raspberry Pi 4 [23] series.

In practice, the front-end services of our solution can record and provide real-time and statistical information about requests and communication latency (see Section 3.2.1). The real-time information about back-end servers can be obtained through Kubernetes Client Library APIs [24]. Information about machine learning models can be profiled in advance or at run time.

#### 4.2.2. Performance under Varying Number of Requests

**Figure 6:**
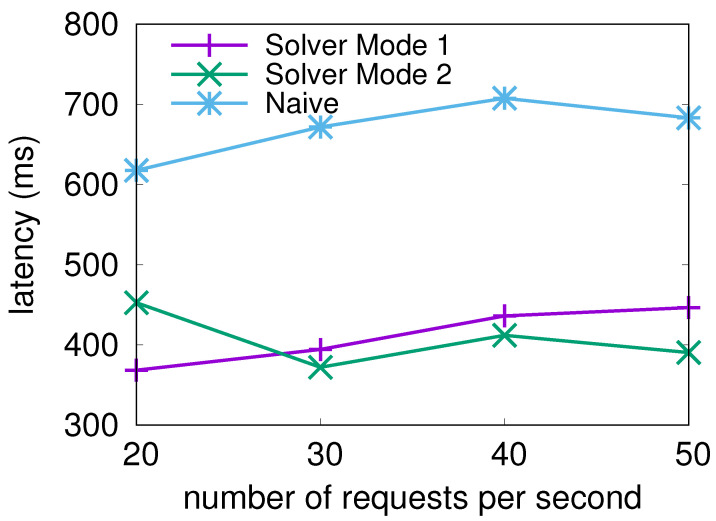
Average serving latency per request while changing the number of user requests per second.

Firstly, we evaluated the average serving latency as the number of requests changed. The spread of request counts represents the number of requests the NLP solver would be asked to consider. Figure 6 depicts the results.

We can see from Figure 6 that in all tests, the average latency achieved by the solver solutions stayed consistently lower than the iterative search solution as the number of requests increased. Furthermore, the average latency remained stable as the number of requests increased, showing that the solver is tolerant of the large shifts in request counts.

#### 4.2.3. Performance under Varying Number of Edge Nodes

Secondly, we evaluated the average latency under a various number of edge nodes. For this test, the number of edge nodes was varied from 5 to 20, similar to the size of an edge cluster in a potential real-world implementation.

**Figure 7:**
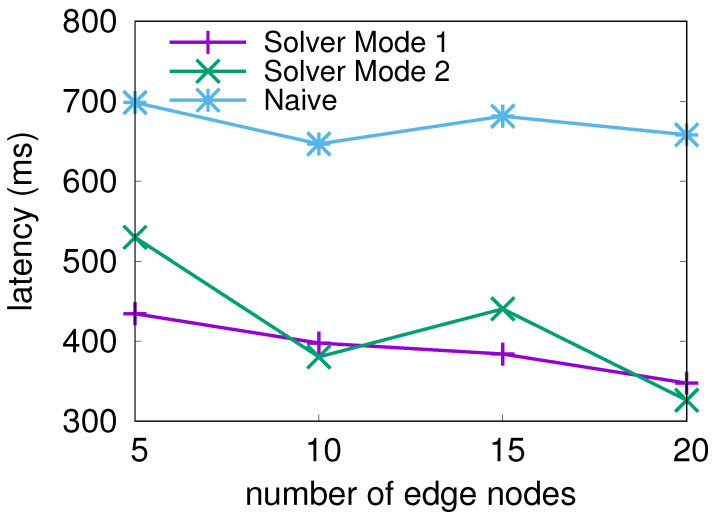
Average serving latency per request while changing the number of edge nodes in the system.

Figure 7 shows us that the average recorded latency value can be seen steadily reducing for all solutions as the number of edge nodes increases. When the number of nodes is increased to 20, the gulf between the solutions increases to 0.32 ms for solver Mode 1 and 0.33 ms for solver Mode 2. In all cases, the difference between solver Mode 1 and solver Mode 2 was within 0.09 ms. These results show how the solver can take advantage of additional computing resources to further reduce the average user-experienced latency.

These simulations show that both solver modes produced a lower average latency result than the iterative search solution. Additionally, both modes produced similar results, showing that either mode is a viable choice when deploying the NLP solver as part of the system.

#### 4.2.4. Solver Execution Latency

Another simulation we conducted was to capture the execution time of the solver to see how efficient it was and how well it dealt with increasing numbers of requests and edge nodes. Table 3 and Table 4 show the execution time of the solver.

These results show that the difference in calculation time overall is negligible. As the number of requests and nodes increases, we can also see that the solver’s computation time is relatively short. This trend demonstrates that even as request or edge node counts increase, the solver will still complete its calculations in an acceptable amount of time.

### 4.3. Discussion

In the experiments, the primary performance metric is the average serving latency, which is also the objective of the optimization problem formulated in Section 2. There could be more performance metrics from the user perspective (e.g., user equipment power consumption) and the server perspective (e.g., server hardware cost). Our solution reduces the overall serving latency experienced by a user, at which point users have the opportunity to turn off their radios between requests and thus save energy. There are, of course, many other factors that could affect user equipment power consumption, but those factors do not directly relate to our proposed solution. Therefore, we did not further investigate how power consumption might be affected by our solution. Our solution optimizes the model provisioning decisions given a certain amount of server resources. When there are fewer server resources available, our solution can still obtain an optimized result. Therefore, we use the average serving latency as the primary performance metric in our evaluation and consider it the best to represent the system performance from the perspectives of both users and servers.

## 5. Related Work

Service placement or model instance provisioning is the core function of the proposed solution. One often-used technique when placing services at the edge is container migration. This technique relies on the layered architecture of popular containers and container runtimes, especially Docker. The core idea behind this technique is to freeze a container, transfer its required components, files, and execution state, and resume it on a new node [8]. The goal is to preserve the current state of the execution while changing nodes and minimize downtime. For example, the authors of [25] proposed a solution for embedded edge computing platforms with service placement by container migration. This solution attempted to minimize the latency caused by service unavailability. It focuses on freezing and migrating models [25] and relies on a cloud orchestrator to continuously issue migration commands. Our solution can reduce the serving time by placing model instances at the optimized edge nodes and significantly reduces the latency caused by migrating containers, a crucial factor for time-sensitive inference jobs.

In [11], the researchers used a time-slot model to allocate services. Users were assigned to a node by the operator in order of arrival. The models can be quickly moved between nodes as users move within the coverage area of the system since a central operator can coordinate mobile users while performing the placement [11]. Our solution does not require continual interaction between an orchestrator and users, which consumes users’ limited bandwidth and energy. Instead, our solution decouples users and back-end services, allowing users to query a front-end and choose a feasible node for satisfactory service. Regarding the reduction of serving latency, [11] requires instant provisioning on the nodes visited by a user. This design may help an individual user, but it will not necessarily benefit all system users. Our solution differs from this strategy by performing optimized provisioning using edge resources holistically to help reduce the average serving latency experienced by users.

The architecture most similar to ours is the uncoordinated access model [10]. In this design, a central orchestrator makes available a pool of edge computing resources in the form of lambdas. The orchestrator also acts as a repository of lambda images that edge nodes can pull from to deploy a new resource. The nodes themselves are tasked with opportunistically choosing which resources to deploy. The system then allows users to choose a resource based on the latency that the user measures. Compared to this solution, our design makes predictive provisioning decisions to optimize the serving latency without also acting as a repository of models. In our solution, each node acquires the models it needs from the Internet and caches them within local storage. This approach enables our solution to perform rapid provisioning and deletion of models per the orchestrator’s instructions.

To reduce the latency on container migration, the authors of [9] experimented with different VM migration schemes that did not require all the data to be transferred before resuming the container. While such a design might be necessary if a job must be moved before completion, we instead focused on minimizing the overall serving latency experienced by users. Our solution creates multiple model replicas, any of which may serve a connected user. Though our approach requires more system memories, it minimizes the serving latency the user experiences by eliminating the extended downtime required for container migration. Our solution is also less dependent on the network speed since the commands to spin up a container require far less data transmission than moving an entire container, its execution state, and the input data of the current job.

Rather than having a cloud orchestrator to issue migration commands, another method is to have the mobile user request a migration itself [12]. This approach allows the user to control migration, thus eliminating the need for the system to continuously monitor each user’s position and make a migration decision. This solution is very close to ours. However, latency spikes are still experienced during the instant provisioning of requested models. Our solution attempts to minimize these latency spikes through optimized provisioning in the back-end.

To summarize, our solution advances the state of serving inference jobs on the edge from the perspective of overall serving latency. It distinguishes from existing solutions in a few key ways. Firstly, the orchestrator (provisioner) in our solution does not monitor and direct how users should move, as its decisions may conflict with users’ movement plans and, in practice, may consume more system resources. We instead use the orchestrator to make proactive model provisioning decisions according to network latency measured at the edge nodes. Secondly, our system creates optimized replicas of a given model on the edge nodes and directs traffic to them while allowing inference jobs to complete wherever they are begun. While this approach consumes more resources than migrating containers, it eliminates the latency and network link saturation imposed by migrating an entire container across nodes. Finally, our solution utilizes an NLP solver to jointly optimize real-time provisioning and inference serving decisions, further reducing the overall latency.

## 6. Conclusions and Future Work

This work presented a novel solution that jointly provisions machine learning models and dispatches inference requests for achieving low-latency inference services on edge networks. We formalized the core problem as a non-linear integer programming problem to minimize the average inference serving latency. Optimization decisions were used to define how and where to place computing resources to minimize the latency experienced by users proactively. We designed and implemented a system-oriented solution with three key modules: the front-end service, dual-mode back-end service, and the NLP solver to perform predictive provisioning operations. Additionally, we also implemented a prototype system to verify the feasibility of the proposed solution on real-world edge computing hardware. Through a series of simulation and testbed experiments, we showed that our solution consistently reduced the inference serving latency compared to a solution that did not perform joint provisioning and request dispatch.

In our implementation, we created the choice for two different operating modes for the back-end service to prioritize short-term latency or long-term system resource balancing. Currently, the choice of which mode to use must be made when the system is invoked. One avenue for future work would be to design improvements to the solution architecture that define how the system could sense the system’s current state and intelligently determine which mode to use during operation. Designing the system to switch intelligently could help avoid overloading edge nodes and directly contribute to keeping the serving latency low. Another direction for improvement would be scheduling the provisioning and deletion of model instances instead of immediately taking action after receiving the solver’s decisions. This way, both the short-term serving latency and long-term system stability can be achieved.

Beyond improving the solution’s performance, an important avenue for exploration is extending the inference jobs supported by the system. The current implementation treats each request as a typical stateless web request. Such an application scenario may fit inference jobs that do not require context or history information, such as those using image or text data as the input. However, for stateful inference jobs such as video data-based inference (i.e., video analytics on edge), simply provisioning multiple model instances over different edge nodes is insufficient. The context of each inference job must also be maintained to ensure the correctness of the inference result. One potential solution is to leverage the optimized model provisioning technique in our solution and combine it with traditional context migration techniques. This way, the inference contexts can be migrated or provisioned together with the required model instances over different edge nodes while users are mobile.

## Figures and Tables

**Table 1:** Testbed provisioning latency.

Test Case	Elapsed Time (ms)
Provision One Model	2548.240
Provision Four Models	11,848.184
Delete Three Models	818.980
Provision One Model and Delete Two Models Simultaneously	3150.921
Provision Three Models and Delete Two Models Simultaneously	9350.82

**Table 2:** Default simulation values.

Parameter	Default Value
Number of Requests per Second	25
Number of Edge Nodes	10
Round-Trip Communication Time	0.1∼0.5 s
Inference Execution Time	0.1∼0.8 s
Size of an Inference Instance	20∼200 MB
Available System Memory	1∼5 GB
Total System Memory	3∼8 GB

**Table 3:** Solver execution time for varied number of requests.

Number of Requests	Mode 1 (ms)	Mode 2 (ms)
20	24.190	20.701
30	25.427	20.569
40	22.583	26.904
50	35.753	34.568

**Table 4:** Solver execution time for varied number of edge nodes.

Number of Edge Nodes	Mode 1 (ms)	Mode 2 (ms)
5	17.153	17.986
10	21.384	19.795
15	22.649	22.786
20	27.405	25.408

## Data Availability

The data presented in this study are available on request from the corresponding author.

## References

[1] Jonas E., Schleier-Smith J., Sreekanti V., Tsai C., Khandelwal A., Pu Q., Shankar V., Carreira J., Krauth K., Yadwadkar N.J. (2019). Cloud Programming Simplified: A Berkeley View on Serverless Computing. arXiv.

[2] Khan W.Z., Ahmed E., Hakak S., Yaqoob I., Ahmed A. (2019). Edge computing: A survey. Future Gener. Comput. Syst..

[3] Wu C., Brooks D., Chen K., Chen D., Choudhury S., Dukhan M., Hazelwood K.M., Isaac E., Jia Y., Jia B. Machine Learning at Facebook: Understanding Inference at the Edge. Proceedings of the 2019 IEEE International Symposium on High Performance Computer Architecture (HPCA).

[4] NVIDIA Corporation Jetson Modules. https://developer.nvidia.com/embedded/jetson-modules.

[5] Reuther A., Michaleas P., Jones M., Gadepally V., Samsi S., Kepner J. Survey and Benchmarking of Machine Learning Accelerators. Proceedings of the 2019 IEEE High Performance Extreme Computing conference (HPEC).

[6] Zhang X., Wang Y., Shi W. pCAMP: Performance Comparison of Machine Learning Packages on the Edges. Proceedings of the 2018 USENIX Workshop on Hot Topics in Edge Computing.

[7] Maheshwari S., Raychaudhuri D., Seskar I., Bronzino F. Scalability and Performance Evaluation of Edge Cloud Systems for Latency Constrained Applications. Proceedings of the 2018 IEEE/ACM Symposium on Edge Computing.

[8] Ma L., Yi S., Li Q. Efficient service handoff across edge servers via docker container migration. Proceedings of the Second ACM/IEEE Symposium on Edge Computing.

[9] Puliafito C., Vallati C., Mingozzi E., Merlino G., Longo F., Puliafito A. (2019). Container Migration in the Fog: A Performance Evaluation. Sensors.

[10] Cicconetti C., Conti M., Passarella A. (2020). Uncoordinated Access to Serverless Computing in MEC Systems for IoT. Comput. Netw..

[11] Ouyang T., Zhou Z., Chen X. (2018). Follow Me at the Edge: Mobility-Aware Dynamic Service Placement for Mobile Edge Computing. IEEE J. Sel. Areas Commun..

[12] Puliafito C., Mingozzi E., Vallati C., Longo F., Merlino G. Companion Fog Computing: Supporting Things Mobility through Container Migration at the Edge. Proceedings of the IEEE International Conference on Smart Computing.

[13] The Kubernetes Authors What is Kubernetes?. https://kubernetes.io/docs/concepts/overview/what-is-kubernetes/.

[14] Pallets Flask Documentation. https://flask.palletsprojects.com/en/2.0.x/.

[15] Wächter A., Biegler L.T. (2006). On the implementation of an interior-point filter line-search algorithm for large-scale nonlinear programming. Math. Program..

[16] COIN-OR Foundation COIN-OR Suite Documentation. https://coin-or.github.io/index.html.

[17] AMPL Optimization Inc. AMPL for Students. https://ampl.com/products/ampl/ampl-for-students/.

[18] TensorFlow-Google Developers TensorFlow Serving Documentation. https://www.tensorflow.org/tfx/guide/serving.

[19] The Kubernetes Authors Kubernetes Documentation. https://kubernetes.io/docs/home/.

[20] kubernetes.io Kubernetes Client Library—Python. https://github.com/kubernetes-client/python/.

[21] NVIDIA Corporation JetPack SDK. https://developer.nvidia.com/embedded/jetpack.

[22] Helmut Hoffer von Ankershoffen Helmuthva/Jetson-Xavier-Tensorflow-Serving. https://hub.docker.com/r/helmuthva/jetson-xavier-tensorflow-serving.

[23] Raspberry Pi Raspberry Pi 4 Tech Specs. https://www.raspberrypi.org/products/raspberry-pi-4-model-b/specifications/.

[24] Kubernetes Access Clusters Using the Kubernetes API. https://tinyurl.com/ywcj9a7d.

[25] Deshpande L., Liu K. Edge Computing Embedded Platform with Container Migration. Proceedings of the IEEE SmartWorld, Ubiquitous Intelligence & Computing, Advanced & Trusted Computed, Scalable Computing & Communications, Cloud & Big Data Computing, Internet of People and Smart City Innovation.

